# Development and Evaluation of An In-House Lumbar Puncture Simulator for First-Year Resident Lumbar Puncture Procedure Learning

**DOI:** 10.7759/cureus.56567

**Published:** 2024-03-20

**Authors:** David Muñoz-Leija, Fernando Díaz González-Colmenero, Diego A Ramiréz-Mendoza, Norma G López-Cabrera, Hilda A Llanes-Garza, Dionicio Palacios-Ríos, Adrián A Negreros-Osuna

**Affiliations:** 1 Radiology Department, Facultad de Medicina y Hospital Universitario “Dr. José E. González”, Universidad Autónoma de Nuevo León, Monterrey, MEX; 2 Anesthesiology Service, Hospital Universitario “Dr. José E. González”, Universidad Autónoma de Nuevo León, Monterrey, MEX; 3 Radiology Department, Hospital Regional Instituto de Seguridad y Servicios Sociales de los Trabajadores del Estado Monterrey, Universidad Autónoma de Nuevo León, Monterrey, MEX

**Keywords:** simulation-based education, spinal anesthesia, simulation design, basic and advanced training in anesthesiology, anesthesiology resident

## Abstract

Introduction: Lumbar puncture (LP) is a common invasive technique considered an essential learning milestone for anesthesiologists due to its application in spinal anesthesia. We aimed to develop an in-house LP simulator, test its effectiveness in learning the steps to perform an LP and analyze its impact on the first-year residents' self-confidence at our hospital.

Methods: We used 3D printing and silicone casting to create an LP simulator based on a lumbar spine computed tomography (CT). We divided 12 first-year anesthesiology residents into control and experimental groups. The control group received traditional training, while the experimental group practiced with the simulator for three months. We used a procedure checklist and a Likert scale survey to evaluate their procedural knowledge and self-confidence at baseline, three, and six months. Eighteen months later, we evaluated their LP performance skills.

Results: Both groups showed a significant improvement in their knowledge scores over time. After three months, the experimental group had a higher median knowledge score (10 (10 - 10) median (min-max)) than the control group (9 (8 - 9.5) median (min-max)) (p = 0.03). While there were no apparent differences in median self-confidence scores between the groups at any time point, the experimental group had a significant increase in their self-confidence for performing an unassisted LP, with a median score of 1/5 (1 - 2.3) at baseline and 5/5 (4.8 - 5) after six months (p = 0.006). In contrast, the control group's self-confidence scores decreased from 4/5 (3 - 4) after three months to 3/5 (2 - 5) after six months. The evaluation of performance skills did not yield statistically significant results.

Conclusion: Our study demonstrates that an in-house LP simulator is an effective and practical approach for first-year anesthesiology residents to learn the LP procedure. This approach could be particularly useful in settings with limited resources and a lack of sufficient patients to practice on, as it provides an opportunity for faster learning and increased self-confidence.

## Introduction

Lumbar puncture (LP) is a common invasive technique considered an essential learning milestone for anesthesiologists due to its application in spinal anesthesia. Konrad et al. describe that an anesthesiologist needs to perform 20 interventions to achieve a 60% success rate in attempts to perform an epidural blockade, which is why it is considered a complex procedure that requires a high level of self-confidence and knowledge to achieve appropriate standards [1]. Simulation-based medical education has proven to be an effective teaching technique for invasive procedures that outperforms traditional methods [2-6]. Its application in LP training can help reduce anxiety in anesthesiology residents as in other specialties [7] while increasing patient safety [8].

3D printing has been effective for the simulation, education, and planning of medical procedures such as ultrasound-guided spinal cord interventions [9,10], surgical planning [11,12], and bronchoscopy [13,14]. Intubation training [15,16], ultrasound-guided spinal insertions [9], and bronchoscopic anatomy models [17] are examples of the applications of this technology in anesthesiology. Although it presents economic and educational benefits, there is scarce literature on its application in this field [18].

We aimed to develop an in-house LP simulator, test its effectiveness in learning the steps to perform LP and analyze its impact on the first-year residents' self-confidence at our hospital.

## Materials and methods

The study design was approved by the local ethical committee under the registration number RA21-00001. All first-year residents of the Anesthesiology Service were invited to participate voluntarily. After obtaining the written informed consent, the included residents were randomly divided into two groups, using number assignment for randomization was performed by number assignment. We collected data at three time points: baseline, three, and six months later. At 18 months after baseline, we evaluated the performance of patients.

Lumbar puncture simulator design and fabrication

Imaging and Segmentation

**Figure 1:**
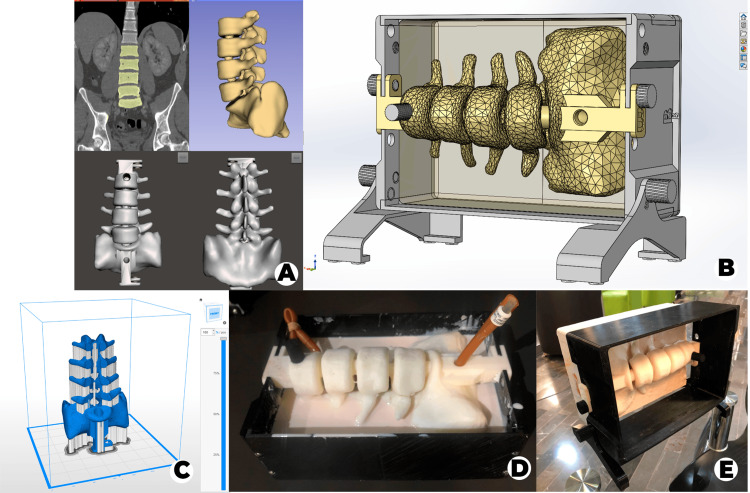
Lumbar puncture simulator fabrication process. (A) Mesh modeling and correction, (B) mold design to simulate skin and soft tissue, (C) model 3D printing, (D) silicone casting, (E) final version.

We selected a lumbar spine CT scan (tube voltage 120 kVp, slice thickness 2.5 mm) with no evident pathology from our database at the Radiology Department. After that, we performed the lumbar column (L2- L5 and half sacrum) and skin segmentation with 3D Slicer version 4.10.2 and exported it as an STL file to correct the errors of the 3D model mesh using Meshmixer version 3.5 (Figure 1A).

Design and Printing

We designed a 25cm x 17cm x 7cm mold from the skin segmentation in SolidWorks 2020 for the silicone casting to simulate skin and soft tissue (Figure 1B), created the 3D printing file with Z-suite version 2.12 (Figure 1C), and printed the model on the Zortrax® M200 with Z-ULTRAT.

Material Preparation

To replicate the ligamentum flavum, we cut a Foley catheter (28 French size) in half lengthwise and fixed it onto the spinal model using screws. Then, to simulate the subarachnoid space, we inserted a Foley catheter (26 French size) filled with water, connected to a 10 ml syringe, into the spinal cavity. We then assembled the mold with the spinal column for silicone casting (Figure 1D). For the casting process, we mixed 3 kg of rubber silicone with 1500 ml of silicone diluent and added 150 ml of catalyst TP (5%) before pouring it into the mold. Finally, we allowed the mixture to dry.

Validation

For validation, a Delphi method was applied, involving a panel of five experienced anesthesiologists who validated the third and final version (Figure 1E) by providing feedback to ensure it met specific criteria. These criteria included palpation of spinous processes, assessment of needle travel sensation, evaluation of ligamentum flavum resistance, and simulation of cerebrospinal fluid outflow.

Lumbar puncture procedure learning and assessment

Initially, both groups did a simulation of LP with our simulator to obtain a basal measurement of their knowledge of the procedure and confidence in its performance, we also asked about how many LPs they performed previously. Knowledge of the procedure was evaluated with a previously reported LP requirements checklist [3], which was adapted by our team (Table 1).

**Table 1:** Lumbar puncture procedure checklist.

Informed consent obtained
Wash hands
Properly position the patient
Know clearly the correct anatomic location for procedure
Put on sterile gloves
Clean the skin with povidone-iodine
Local anesthesia; using 1% lidocaine
Correct use of needle gauge
Slowly advancing toward the midline insertion, equidistant to the L3-L4 spinous processes
Knowledge of the opening pressure with the patient in the supine position (normal values being 180 mmH_2_O in adults and up to 200 to 250 mmH_2_O in obese adults)

The baseline perceived confidence of included residents on their knowledge for performing LP under supervision and unsupervised was measured with an online-based Likert-type survey with three items and five possible levels of confidence: 1) not at all confident, 2) not very confident, 3) unsure, 4) fairly confident, 5) very confident.

Afterward, the residents attended a class given by experts belonging to the anesthesiology service, where the procedure to perform an LP was thoroughly explained, based on our adapted LP requirements checklist. The control group continued with the traditional method (i.e., attending further classes and performing LPs under expert supervision), while the experimental group complemented their training with the continuous use of the simulator until they achieved at least 30 simulated procedures for 3 months. We repeated the measurements of knowledge and confidence 3 months after the basal measurement and 6 months after the basal measurement.

Eighteen months after the intervention, we assessed the performance of both groups by evaluating specific criteria in 10 patients from each group. The criteria evaluated included procedure time (from the start of local anesthesia administration), the number of attempts made, and whether a redirection was needed.

Statistical analysis

Baseline demographic characteristics (age, gender, previous experience, and number of previous LP) were described with means ± standard deviations (SD) and frequencies and percentages and were compared using Mann-Whitney’s U for numerical variables, and Fisher’s exact test for categorical variables. All the numerical results derived from the scales were described with medians and interquartile ranges (IQR).

Procedure knowledge test scores of the overall population, obtained at baseline and after three and six months of follow-up were compared using the Friedman test, if a significant p-value was obtained in the omnibus test, a post-hoc Wilcoxon signed-rank test was performed between each assessment time.

The numerical scores from the Likert scales of the two groups at each measurement period were compared with Mann-Whitney U tests. The totality of the post-graduate year (PGY) 1 residents in the anesthesiology program of our hospital were included as the sample for this study. To calculate our study's sample size, we conducted a two-sample means test targeting an 80% power and a 0.05 alpha level. This aimed to detect a 2-point difference in procedural knowledge test scores between a control group (mean = 8) and an experimental group (mean = 10), with assumed equal standard deviations (SD = 1) across groups. Our analysis concluded that 12 participants, divided equally into six per group, were necessary to meet these statistical requirements.

A p-value threshold of <0.05 was considered as indicative of statistical significance, all statistical analyses were performed on Stata Statistical Software (StataCorp. 2019. Stata Statistical Software: Release 16. College Station, TX: StataCorp LLC.).

## Results

Model development

The total cost for the materials used in the LP simulator development amounted to 300 USD. While the study utilized a Zortrax® M200 3D printer, this was a pre-existing equipment in our laboratory. However, for reference, the Zortrax® M200 printer is priced at approximately 2800 USD.

Model effect in resident LP procedure learning

**Figure 2:**
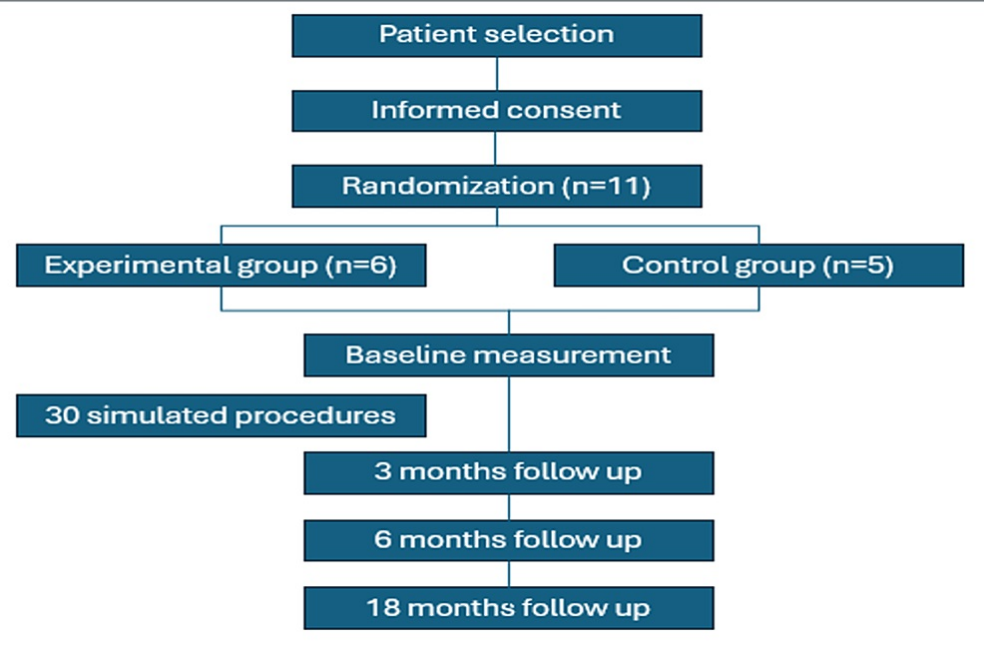
Study flowchart.

Out of the 12 PGY-1 residents initially considered, we excluded one participant due to residency desertion, the rest of the participants were mostly males compromising 63.6% (n = 7), aged between 26 and 30 years. The study flowchart is shown in Figure 2.

We randomly divided the participants into a control group (mean age 27.6, ± 2.2, one woman and four men) and an experimental group (mean age 27.7, ± 1.6, three women and three men). There was a higher proportion of residents with previous experience performing LP in the control group 60% (n = 3) than in the experimental group 33.3% (n = 2), however this difference did not reach statistical significance (p = 0.567). The mean number of previous LP in the control and the experimental group were 0.8 and 0.7, respectively (Table 2).

**Table 2:** General demographic information and procedure knowledge results A p-value <0.05 was considered statistically significant. FU: follow-up; IQR: interquartile range; LP: lumbar puncture; SD: standard deviation *Significant post-hoc difference between baseline vs 3 months FU, and baseline vs 6 months FU.

	Total population (n = 11)	Experimental group (n = 6)	Control group (n = 5)	P-value
Age, years (mean ± SD)	27.6 ± 1.8	27.7 ± 1.6	27.6 ± 2.2	0.931
Females, n (%)	4 (36.4)	3 (50)	1 (20)	0.545
Previous LP experience, n (%)	5 (45.5)	2 (33.3)	3 (60)	0.567
Procedure knowledge test – median (Interquartile range (IQR))				
Baseline	6/10 (6 – 7)	6/10 (5.8 – 7)	7/10 (5.5 – 7)	0.662
3 months FU	10/10 (9 – 10)	10/10 (10 – 10)	9/10 (8 – 9.5)	0.03
6 months FU	10/10 (9 – 10)	10/10 (9.8 – 10)	9/10 (9 – 9.5)	0.08
Intraclass difference p-value	<0.001*	0.003*	0.02*	-

Procedure knowledge test

The procedure knowledge test showed a significant improvement over time in the overall population (p < 0.001), with a median baseline test score of 6 (6 - 7), and a median score of 10 (9 - 10) after three months, which was maintained after six months. Post-hoc tests indicated a significant difference between baseline, three (p = 0.003), and six (p = 0.003) months scores. When analyzed separately, both groups showed a significant improvement in their knowledge scores over time (p = 0.003, for the experimental group, and p = 0.02 for the control group).

When comparing both groups in each time period, we only found a significant difference between the knowledge scores after three months (p = 0.03), with a median score of 10 (10 - 10) in the experimental group, and of 9 (8 - 9.5) in the control group. Additional information on the scores at different time points can be found in Table 2.

Knowledge self-confidence survey

The results of the self-confidence survey can be found in Table 3.

**Table 3:** Self confidence survey results A p-value <0.05 was considered statistically significant. FU: follow-up; IQR: interquartile range; LP: lumbar puncture *Significant post-hoc difference between baseline vs 3 months FU, and baseline vs 6 months FU

	Total population (n = 11)	Experimental group (n = 6)	Control group (n = 5)	P-value
Knowledge self-confidence – median (IQR)				
Baseline	1/5 (1 – 2)	1.5/5 (1 – 4)	1/5 (1 – 1.5)	0.33
3 months FU	4/5 (4 – 5)	4/5 (4 – 5)	4/5 (4 – 5)	0.79
6 months FU	5/5 (4 – 5)	5/5 (4.8 – 5)	4/5 (3 – 4)	0.25
Intraclass difference p-value	<0.001*	0.013*	0.012*	-
Confidence in performing LP with assistance – median (IQR)				
Baseline	2/5 (1 – 4)	3/5 (1 – 4.3)	2/5 (1.5 – 3.5)	0.79
3 months FU	5/5 (5 – 5)	5/5 (5 – 5)	5/5 (4.5 – 5)	0.66
6 months FU	5/5 (5 – 5)	5/5 (5 – 5)	5/5 (4.5 – 5)	0.66
Intraclass difference p-value	<0.001*	0.007*	0.012*	-
Confidence in performing LP unassisted – median (IQR)				
Baseline	1/5 (1 – 2)	1/5 (1 – 2.3)	1/5 (1 – 1.5)	0.66
3 months FU	4/5 (4 – 4)	4/5 (4 – 4.3)	4/5 (3 – 4)	0.43
6 months FU	5/5 (3 – 5)	5/5 (4.8 – 5)	3/5 (2 – 5)	0.18
Intraclass difference p-value	<0.001*	0.006*	0.05	-

A significant increase in the knowledge and self-confidence was observed in the general population (p <0.001). At baseline, most of the residents felt insecure with their knowledge for performing a LP, with 63.6% (n = 7) reporting a 1/5 confidence level, during the study their confidence level increased with 72.7% (n = 8) reporting a 4/5 confidence level after 3 months, and 63.6% (n= 7) reporting a 5/5 confidence level after 6 months. Both study groups showed a similar significant increase in their knowledge/self-confidence across the follow-up evaluations, with no significant differences in the knowledge self-confidence scores at any time point. At the end of the study, more residents had confidence in their knowledge for performing a LP in the experimental group 83.3% (n = 5) than in the control group 40% (n = 2), however, this difference did not reach statistical significance (p = 0.28).

Confidence in Performing a LP with Assistance

When asked about their confidence in performing a LP on a patient with the assistance of a professor or senior resident, a similar proportion of participants felt totally unconfident 27.3% (n = 3), somewhat unconfident 27.3% (n = 3), and somewhat confident 27.3% (n = 3) in their abilities. After three months, 90.9% (n = 10) were totally confident in their abilities for performing an assisted procedure, this result was maintained after six months. The results of the survey are described in Table 3, both groups showed a significant increase in their scores during follow-up, however, no significant differences between the scores of the groups were found at any time point. At the end of the follow-up, most of the residents of both groups had a 5/5 confidence level in their abilities for performing an assisted LP (100% (n = 6), experimental; 80% (n = 5), control group).

Confidence in Performing a LP Unassisted

When asked if the subjects were confident in their ability for performing an unassisted LP, 72.7% (n = 8) felt insecure at the baseline assessment. After three months practicing with the model, 81.8% (n = 9) felt somewhat confident in their abilities, their confidence improved at the end of the follow-up with 63.6% (n = 7) reporting feeling perfectly confident in their abilities for performing an unassisted LP. The results of the survey are described in Table 3. Only the residents in the experimental group showed a significant improvement in their survey scores, notably, after six months the residents in the control group showed a reduction in their survey scores, however, no statistically significant differences were apparent between the experimental groups.

Performance Evaluation

When assessing the duration required to execute a successful spinal anesthesia, the mean time was 5.7±3.1 minutes overall. The overall mean number of attempts to achieve a successful spinal anesthesia was 1.4±0.2. Redirection was necessary in 45.5%±14.4 of cases overall. We did not find significant differences between the experimental groups. For a detailed analysis of the findings, please refer to Table 4.

**Table 4:** Performance evaluation results A p-value <0.05 was considered statistically significant.

	Total population (n = 11)	Experimental group (n = 6)	Control Group (n = 5)	P-value
Procedure time (minutes) – (mean ± SD)	5.7 ± 3.1	4.7 ± 0.7	6.8 ± 4.5	0.28
Number of tries – (mean ± SD)	1.4 ± 0.2	1.4 ± 0.3	1.3 ± 0.2	0.52
Percentage of times redirection was necessary – (mean% ± SD)	45.5% ± 14.4	43.3% ± 15.1	48% ± 14.8	0.62

## Discussion

The potential benefits we found of our simulator are the following: patient-based anatomical fidelity, longer use-time potential (i.e., one can replace materials easier than in commercial models), avoidance of cadaver usage issues (i.e., ethics, storage, disposal, or inconvenience of use), customizability based on patient characteristics (e.g., obesity, anatomical malformations) or for a preoperative evaluation. Previous studies have shown comparable fidelity between commercial and home-built simulators [10]. Our results indicate that our achieved effectiveness and user satisfaction are comparable to ones achieved with commercial simulators, further supporting the benefits of these simulators. Our simulator focused on offering vital aspects of the LP procedure. The experts validated the ligamentum flavum resistance loss and the palpation of the spinous processes and intervertebral spaces. The only feature they disagreed with was the simulator's skin texture.

Our findings were similar to reports in other specialties. McMillan et al. showed that using a LP simulator combined with an interactive didactic session significantly improves procedural competence (from 35.7% - to 68%) and decreases self-reported anxiety in PGY1 pediatrics residents [7]. Similarly, Sun ad Qi found that LP simulators increased students' self-confidence after the intervention [4]. Barsuk et al. reported an increase in the average LP checklist procedure from 46.3% to 95.7% when applied in PGY-1 internal medicine residents, while PGY2-4 neurology residents averaged 65.4% with the traditional method [3]. These results, alongside our own, might indicate that training with simulators is a more effective learning technique than traditional shadowing methods. Moreover, the adequate application of these simulators could prove less economically burdensome than commercially available ones. In comparison to commercial LP simulators, which can cost as much as 4800 USD, our method offers significant cost savings once the initial investment in a 3D printer is made. With a 3D printer already available, the expense of creating additional simulators or other teaching tools dramatically reduces to the cost of materials alone. This approach not only presents a cost-effective alternative for educational institutions but also emphasizes the versatility and economy of in-house fabrication using 3D printing technology.

In this randomized study, we found that the use of a LP simulation model is an effective tool for learning the correct LP procedure. Regarding self-confidence, when performing the procedure, we observed an increment in baseline confidence levels in the experimental group, however, this difference did not reach statistical significance, possibly due to the limited number of subjects. Furthermore, during our fourth measurement, we were unable to detect any significant differences between the groups. This outcome may be attributed to the time interval between the third and fourth measurements. It is worth noting that our hospital provides ample opportunities for practice, which could lead to increased experience among participants by the time of the fourth measurement, potentially offsetting the effects of the intervention.

Mason and Strike found that 42% of doctors felt inadequately trained to perform a practical procedure safely when first performing it alone with the traditional “see one, do one, teach one” approach [19]. Medical practice may differ in institutions due to the number of patients they admit and the absence of people with certain diseases. For this reason, some doctors tend to take longer to develop self-confidence and, ergo, to increase the number of successful interventions, which simulators as an alternative to making medical procedures can strengthen. We also underscore that building one with accessible materials can be cheaper than acquiring commercial models and firmly believe that combining this technology with the traditional approach could offer high-quality training.

Our study had some limitations. Although we included the totality of PGY1 anesthesiology residents in our institution, the sample was small. Moreover, we could not blind participants due to the intervention explanation during informed consent. On the other hand, our simulator model was validated only by expert anesthesiologists working at our hospital, as no external validation panel was available. Ultimately, although the materials used had some similarities to human tissue, they did not entirely mimic the required sensitivity necessary for the procedure. Utilizing in-house-built simulators could provide a cost-effective alternative to commercially available models. This could help to increase access to training and improve procedural competence among healthcare providers in areas with limited resources.

## Conclusions

Using an in-house-built LP simulator as a training tool can accelerate the acquisition of all the necessary steps, as compared to the traditional method, leading to increased confidence when performing the procedure. This technique offers a cost-effective and accessible alternative, particularly in regions with limited healthcare infrastructure. By improving access to training, these simulators have the potential to enhance procedural competence and improve patient outcomes in settings with limited resources.
